# Dr. Monro on Hydrocephalus

**Published:** 1828-04-01

**Authors:** 


Medico-Chirurgical Review,

No. XVI.

[FASCICULUS V.]

MARCH 8, 1828.

### Art. XII.

*The Morbid Anatomy of the Brain.* By Alexander Monro, M.D. &c. &c. Vol. 1. *Hydrocephalus.* 8vo, pp. 200, with five coloured plates. Edinburgh and London, 1827.

Whether we reflect on the mortality occasioned by hydrocephalus, or on the learning and opportunities for observation of the author, we cannot but conclude, that the volume under review will attract considerable attention. Dr. Coindet has stated that twenty thousand deaths annually result from hydrocephalus in France—while Dr. Alison informs us that 40 out of 120 patients die of this disease in the New Town Dispensary. According to the late Dr. Davis, of London, 8 out of 45 deaths, in the Universal Dispensary, were produced by hydrocephalus.

In respect to the author, it appears that it is now more than twenty-five years since he began to direct his attention to organic disorders of the brain—but, after long observation and patient investigation, he had nearly given up the undertaking, from the difficulties of the subject, when the death of a relation, by a species of hydrocephalus, to him quite novel, gave a new impulse to his researches, and enabled him to accumulate the mass of facts herewith presented to the public. We shall do most justice to the author, and confer most benefit on our readers, by confining ourselves, in this article, almost entirely to the task of analysis, since the facts are already in a very concentrated state, as well as in very great number.

The work is divided into two chapters—one containing the morbid anatomy—the other the symptomatology, &c. of hydrocephalus. We shall prefer reversing the order which Dr. Monro has chosen, and take his second chapter first. This second chapter, indeed, of itself, offers ample scope for an analytical article. We shall follow the sections seriatim.

Sect. I. *Hydrocephalus, in which the Skull retains its usual Size and Form.*

Of this, there are two very different species—the one decided in its character at the outset—extremely rapid in its progress, proving fatal in three, four, or five days—very rare in its occurrence, and, according to our author, undescribed by any other writer. The other species is obscure in its origin—slower in its progress (being of three or four week's duration)—very frequent in its occurrence—and described by many writers.

MOST ACUTE SPECIES.

"This rare form of the disease is very sudden in its attack. There is no previous headach, drowsiness, stupor, nausea, vomiting, paralytic state of any part of the body, or any one symptom denoting a derangement in the functions of the nervous system.

"It begins like the croup. The child awakes in the night in a state of extreme agitation, and much flushed, and with a quick pulse ; he is hoarse, and the sound of the voice when he inspires is similar to that in croup,—the sound seems to come from a brazen tube, which is contracted at a certain part.

"Children who are stout and healthy, are equally liable to this disorder as the feeble and emaciated. And I have seen a patient, on the very day he was attacked by this disorder, who seemed very cheerful, and took his meals well, and was to all appearance in the most perfect health.

"The giving an emetic relieves the breathing, and, upon examining what has been rejected by vomiting, it is found to be evidently undigested."*

Some cases are given by Dr. M. in illustration of this very fatal disease.

*Case.* At half-past ten o'clock, P. M. (Sunday) a stout boy, 20 months old, awoke suddenly from his sleep, (which had been apparently tranquil) as in a fright, and coughed violently, with a croupy sound, his breathing being difficult and quick. He seemed feverish—was thirsty—unwilling to lie down—pulse 150 in the minute, and rather hard. He had been in apparently perfect health during the preceding day, which was intensely cold. He had had two natural motions, and ate his food heartily. Under the impression that the disease was common inflammatory croup, an antimonial emetic was administered, which operated mildly, and brought up some ropy mucus, bile, and *undigested* turnip and carrot, eaten the preceding day. Mr. Bryce and Dr. M. saw him a quarter before 12, when an ipecacuan emetic was exhibited, and afterwards the semi-cupium. When the vomiting subsided, three grains of calomel were given, and the child fell into a sleep at 4 the next morning, when a large blister was applied to the larynx, but slipped down on the sternum. At 8 A.M. (Monday) a dose of castor-oil was given to work off the calomel. Three offensive stools, one of

* "When this sheet was about to be sent to press, I learned from Professor BURNS that he had described, in his Principles of Midwifery, this very acute form of hydrocephalus, which he attributes to an affection of the origin of the eighth pair of nerves, induced by the state of the extremity of the fifth in dentition acting on its origin, which is near the eighth. The recurrent seems more immediately in fault, producing a temporary paralysis of the muscles of the glottis rather than a spasm. It by no means ends necessarily in hydrocephalus, but it sometimes does, as, after the child has been apparently well for weeks or months, he is carried off by hydrocephalus, which change is first indicated by general convulsions. Few children recover when the original attack is accompanied with convulsions, yet the case is not altogether hopeless."

them green, were discharged. At 9 o'clock that morning, Dr. M. and Mr. Bryce found the child more lively, his breathing less frequent, and his voice stronger. He now sang and laughed, but his voice was still husky. At 4 o'clock that day, feverish symptoms came on, and his breathing was more affected. Tepid lavations and a dose of calomel were prescribed, and he passed a tolerable night. *Tuesday morning* found the little patient in nearly the same state; but in the evening, there was another exacerbation of fever, cough, and difficulty of expectoration. At 10 P.M. the pulse was 200, and very hard and sharp. He breathed rather like an asthmatic than a croupy patient—countenance much flushed, and indicative of great distress. Mr. Bryce examined the child's mouth, and found the gums much swelled and inflamed, and the dentes caninæ, on each side, about to cut the gum. The gums were incised freely, and there was much discharge of blood. The child now started much more frequently than before. Five leeches were applied to one of the legs, and much blood was evacuated by means of immersion in warm water. Three grains of calomel were given, and procured a large healthy evacuation. We need not pursue the details any farther. The child died the next day (Wednesday) at 5 P.M. The dissection is very interesting, and we shall give it in the words of the author.

"*Dissection* 12 *hours after death.* The cavity of the abdomen being first examined, the intestines were found in their usual situation, of a white colour, and moderately distended with air. The stomach, which was much contracted, lay in the left hypochondriac region, exhibited a blanched appearance; and, when slit open at the lesser curvature, it was found empty, and its mucous membrane was uniformly healthy, and the rugæ were very distinct. The mesenteric glands were of their natural colour, size, and consistence, and all the abdominal bowels. The large bloodvessels also were almost empty.

"All the bowels of the thorax were sound.

"The veins on the fore-part of the neck were quite empty; the trachea externally was of a silvery whiteness. An incision was made from the upper part of the thyroid cartilage into the division of the wind-pipe; the mucous membrane lining the larynx was of a white colour, and in every respect healthy. There was no appearance of the croupy membrane, no thickening, no ulceration, nor any mark of preceding inflammation. The trachea and branches of the windpipe, as far as they could be traced, were also healthy and free of mucus. The fauces and pharynx were also sound.

"The *brain was large, firm, and healthy;* and the veins entering the sinuses were distended with dark blood. *The upper surface of the brain, particularly the superior part of the posterior lobes, was covered with a transparent gelatinous effusion. On opening the ventricles, about an ounce of colourless serum was discovered, which had raised the fornix considerably.* The medulla oblongata and tuber annulare were found floating on a great quantity of clear serum. The veins covering the tuber annulare and medulla oblongata were distended with blood, so that they exhibited a deep scarlet colour. All the nerves at their origin were sound, *except the fifth and eighth pairs, which were also of a deep scarlet colour, and covered with turgid vessels.*

"*On removing the brain, by cutting through the medulla oblongata, a considerable quantity of serum rushed from the upper part of the spinal canal.*

"The vessels of the spinal marrow were turgid, those at the cervical portion of a vermilion-red colour, and those of the lumbar portion of a dark red hue. *The eighth pair of nerves was of a deep uniform red colour along its whole tract, as far as its branches going to the lungs.*"

The author proceeds to the particulars of some other cases of this formidable disease, which is not confined to the infantile state. Thus, a woman, aged 65 years, was subject to spasms in her stomach, and obstinate constipation. She died very suddenly. On dissection, no morbid appearances could be found in the stomach or intestines. "There was considerable effusion of a watery liquor, and also distention of the vessels of the pia mater over the eighth pair of nerves within the membranes, and at the base of the brain." The following case occurred in the practice of Drs. Monro and Saunders.

"J. B., æt. eight months, a plump stout child, became remarkably fretful, and would take no kind of food. Next day he was seized with slight cough and hoarseness, which gradually increased, and also with difficulty in swallowing; so that a part of his food, upon his endeavouring to swallow, was rejected through his nose. Pulse quick.

"On the following day, the symptoms had materially abated, especially the cough, and he expectorated a good deal of viscid phlegm.

"During the night, and still more during next day, he became much more uneasy; his breathing became more oppressed; and the voice was so hoarse and shrill, that several old women who saw him, said *he had the croup*. There was great heaving and agitation of the chest.

"On the subsequent day, the breathing was very laborious, and much hurried; pulse very rapid, and between 120 and 130 in the minute; and next day he died.

"*Appearances on Dissection.*—Skull very vascular. There was a slight effusion of serous fluid between the arachnoid coat and pia mater; and about ℥i. of a similar fluid was contained within the ventricles of the brain. *The vessels of the pia mater at the corpora quadrigemina and tractus optici, and at the origin of the eighth pair of nerves, were much distended with blood.*

"Lungs quite sound.

"No morbid appearance was discovered in the larynx and trachea. A small quantity of mucus, which had more of a greenish hue than in the sound state, ran out on opening the windpipe, and there was some frothy mucus within the smaller branches of that tube.

"The only morbid appearance in the abdomen was a very considerable enlargement of the mesenteric glands.

"The symptoms in the preceding cases corresponded in my opinion with the phenomena which occur upon irritating the eighth pair of nerves of the inferior animals.

"The croaking sound of the voice, and the difficulty in breathing, were probably to be imputed to the effects of the irritation, to which the laryngeal nerves had been exposed, for the stretching or dividing these nerves in a living animal produces the same effects.

"The functions of the lungs and stomach are suspended; from the irritation applied to the eighth pair of nerves, which also happens when these nerves have been divided. If the eighth pair of nerves of an animal be divided, and if that animal be allowed to take food soon after the division of the nerves has been made, vomiting in a short time ensues. Precisely the same took place in the child above described,—and the food is found to be unchanged,—which also happens in this form of hydrocephalus. In the case above mentioned, the vegetable substances which the child had taken in broth, *were indigested, though they had been within the stomach for twenty hours.*

"In the child's case detailed in page 70, &c. the difficulty in breathing gradually increased, and the face became purple towards the conclusion of the disorder; and, upon examination after death, the air-tubes and cells of the lungs were found filled with a frothy fluid; and hence the lungs did not collapse. All which circumstances take place when the eighth pair of nerves of an animal have been divided.

"In the cases above detailed, the difficulty in breathing was much increased by the effort of vomiting; and the same happens when the eighth pair of nerves of an animal have been divided."

Another case is quoted from the work of Mr. Swan on the nervous system, which bears considerable analogy to the above.

### *Diagnosis of this Hyper-acute Form.*

It is of some importance to be able to distinguish this dangerous modification of hydrocephalus from the more common forms of croup. It is not, according to our author, dependent on the state of the weather, the locality of residence, or on any of the predisposing causes of croup ; " but is rather connected with the period of teething, and nervous irritation." The following are the more remarkable distinctions between this disease and the common croup.

" The patient, at the outset of the disease, seems in a state of nervous irritation, often starts in his sleep, and in a short time the disease assumes the appearance rather of a spasmodic affection of the larynx, than of the inflammatory croup ; but there is not so much wheezing, and the disorder is not mitigated by the expectoration of ropy mucus, or by vomiting ; and what is discharged by vomiting may be observed to be indigested.

" The purple colour of the face is a striking feature of the earlier part of the croup, but it is not obvious in this disease until twelve or fourteen hours before death, and is probably owing, as in moribund animals, to a weakness of the muscles of inspiration.

" The longer the duration of this disease, the less shrill and hoarse the voice becomes ; whereas in the common croup the contrary takes place.

" Coagulable lymph sometimes forms a distinct tube within the larynx and windpipe in the advanced stage of common croup, and may be perceived moving within it, which never happens in this disorder.

" This disorder may be readily distinguished from inflammation of the lungs ;— there is no cough, or pain in any part of the side or breast, which is increased by inspiration ;—in proof of which, the first mentioned patient repeatedly sang during the progress of the disorder, and as loudly as when he was in the most perfect health. This form of hydrocephalus may be distinguished from the more common form of the disease; as the sight is not impaired, and there was no dilatation of the pupils, and little or no delirium.

" Lastly, When the voice becomes weak or shrill, from any cause compressing the windpipe,—as by matter collected behind it,—by diseases of the bronchial or thyroid glands,—by aneurism of the aorta, or carotid artery,—or by any other cause compressing and straitening the windpipe, the alteration from the natural sound of the voice is permanent ; whereas, from this disorder, it is but temporary."

The method of treatment may be soon dispatched. The rapidity of the disease, and the seat of the effused fluid, leave little to be done by art. The exhibition of a large dose of calomel, and the application of numerous leeches to the head, with a blister to the nape of the neck, at the very commencement of the attack, seem to Dr. Monro the most proper remedies.

We now proceed to the more common form of the disease.

### ACUTE HYDROCEPHALUS.

It must be confessed that there is not one pathognomonic symptom of a collection of water within the brain. The characters of the disease are deduced rather from a chain or combination of symptoms—and hence the nature of the disease remains often undetected until it is beyond the reach of art, unless the most scrupulous attention be paid to the most minute phenomena. The following graphic description of the early symptoms which characterise hydrocephalus, is deserving of record in this place. We could not abbreviate without injuring them.

"The earlier symptoms are those of irritation, the latter those of oppression.

"The early symptoms are generally so mild as to attract but little attention, and often escape the notice even of the parents of the patient. The disease is most frequent in persons of a scrofulous constitution, where there is little energy of the system, and little activity in the vascular system; or, it is either the consequence of some organic disorder of the brain, or its investing membranes,—or of such disorders as impede the free return of blood from the head, or the free circulation through the bowels of the chest.

"The early symptoms of the more mild form of the disease, are, a sense of general uneasiness over the body, accompanied by languor, paleness, and collapse of the features; the eyes look languid, have not their usual brilliancy, and frequently are turned upwards; the child is peevish, which is strongly expressed in his countenance; the countenance has no longer the bloom of health; his head feels heavy, and the child, unwilling to run about, seems easily fatigued, and always desirous of lying down on the top of the bed. His appetite is bad, and capricious—and seems unwilling to take the trouble of eating, and there is often bilious vomiting.

"If the child be old enough to express his feelings, he complains rather of a dull noise in his head than of acute pain, and this is increased somewhat on pressure. He is very restless in bed, and seems much disturbed in his sleep.

"In a short time the symptoms become more urgent, the skin becomes very dry, and the lips are cracked. There is a permanent lassitude; the bowels become torpid; the child complains of constant pain over the eyebrows, which are swollen, and of giddiness; nausea and sickness succeed. There is a total loss of appetite. The senses of vision and hearing are morbidly acute; the light is intolerable,—the patient turns away from it,—keeps his eyelids half-closed,—is awakened from his slumber by the slightest noise, and often starts from sleep screaming. He will not allow his pulse to be felt.

"Many children suffer very little, if at all, from headach, at the commencement of the disease; and in those who have, we observe that there are remissions.

"The headach is relieved in the earlier stage of the disease by purgatives.

"The cheeks are much flushed, especially in the evening, and one cheek is frequently more flushed than the other; his nose is very itchy, and his tongue hot and dry. The patient awakes generally in a fright, and screaming; seeming to suffer excruciating headach, to relieve which, he clasps his hands on his head; even when not disturbed, he sleeps only for two or three hours at a time; he suffers from sickness, expresses disgust for every kind of food, and vomits especially when moved; and, as Doctors Fothergill and Quin have well observed, 'the sickness and headach sometimes alternate with each other.'

"These symptoms are accompanied by heat of the skin, and quick pulse, amounting to 100 or 110 in the minute, and but rarely by thirst.

"One of the most striking features of this disease, is a torpid state of the system;—salivation is not readily excited; nor does a blister rise well; and very considerable doses of antimonial powder do not occasion even a slight degree of moisture on the skin.

"The patient is costive generally, and is not easily moved by a purgative medicine.

"The fæces have an oily appearance, sometimes a very fetid smell, are generally of a green or brown colour, somewhat like chopped spinage—which is not owing to calomel, as it is observed when no calomel has been given, and are in consistence somewhat like glue. Towards the conclusion of the disease, diarrhœa sometimes comes on to such a degree, that opium is required to check it.

"The urine is sometimes retained for a longer time than during health, and there are also instances in which the little patient had a desire every hour to pass water;—and I attended a child afflicted by this disorder, who passed for some days very little urine; but within four or five days of his death, he passed fully as much urine as when in perfect health. Often the power of expelling the urine is completely lost, so that the regular use of the catheter is required.

"The position and gestures of the child in bed, and the effects of motion, merit attention, being very characteristic of the disease.

"A child with this disease cannot bear the erect position, or to be moved evidently from the headach he suffers; he is tolerably easy only when in bed, and excluded from light. In the earlier part of the disease, he cannot sleep with the head low; he lies in bed with outstretched arms, which have a tremulous motion—are often directed towards the head, or firmly clasped upon it; he is constantly turning and tossing from one side of the bed to the other, as if he could not find an easy posture, and generally kicks the bed-clothes with one or both feet, and very frequently groans much, as if under the influence of pain.

"The gait of the patient is peculiar; he totters, and cannot walk with his usual firmness, one side of the body being weaker than the other; he lifts his legs very high, and takes long steps of unequal length, like a paralytic person. This is a bad symptom, and generally connected with disease at the base of the brain.

"In the progress of the disease, the sleep is still more disturbed, and the breathing becomes more irregular and difficult. The breath, according to Dr. WHYTT, has a sickish and most offensive smell, which he never had observed in any other disorder.

"There are also generally symptoms of a derangement in the functions of the alimentary canal. Dr. CARMICHAEL SMYTH has remarked, 'Their abdomen is commonly flat, and compressed in a manner I have seldom observed, except in persons affected with the painter's colic.'

"The tongue is foul; sometimes it has a shining red appearance, or it is covered with aphthæ.

"There is much variety as to the severity of the symptoms, which merits much attention, as demonstrative of the cause of the disease, its seat, and as regulating the prognostic, and mode of treatment.

"Dr. ABERCROMBIE has observed, when 'the effusion was combined with that peculiar destruction of the central parts of the brain, which I have given my reasons for considering as the effect of inflammation of these parts, there has been severe and deep-seated pain.'

"The combination of the above symptoms, the morbid sensibility as to light and hearing—great irritability of temper—the head hotter than the rest of the body, and often covered by a clammy perspiration, sufficiently mark this disorder in its early stage.

"After the disease has been of a few days duration, the pulse, which had been quick and irregular, sometimes becomes slower than in health, and also irregular; which, according to Dr. WHYTT, marks the second stage of the disease."

The second stage need not occupy us long. In this stage, the pulse is slower than in the first, or even than in health, besides being unequal and irregular. The skin continues hot, or even hotter than before, while most of the symptoms above enumerated increase in severity. There is a want of correspondence in the movements of the eyes in this stage, and some degree of strabismus may be observed, while the patient's sufferings are evidently rendered more acute. He groans more frequently—gnashes his teeth—loathes every kind of food—sometimes has violent vomiting, while the head-ache is so acute as to occasion loud screaming. About this period, some patients experience a temporary remission of the more marked symptoms, from which the friends—and occasionally the medical attendants, draw false conclusions.

*Third Stage.*

This may be termed the stage of collapse. Irritability changes into stupor and drowsiness, and the body often becomes more cold than natural. The pulse is now very rapid—sometimes amounting to 200 in the minute.

The duration of this stage is very various—from ten or twelve hours to several days. The child is unable to turn the head or move the body—he sinks down in bed—is very drowsy, and often insensible to all irritations. One side of the body is frequently paralytic, which is a bad symptom. The eye-lids often droop, to a certain extent, over the pupil, and, on raising them, the cornea appears muddy, and the pupil is much enlarged. The sight is indistinct or double —indeed all the senses, except that of hearing, appear blunted. Emaciation proceeds rapidly. Sighing is very common. The discharge from blisters applied to the head is remarkably fetid. The pupil is sometimes dilated and insensible to light—sometimes contracted—which is equally a proof of extreme blindness and insensibility of the retina. Delirium is a frequent symptom, at this early period of the disease; and comes on much more suddenly than in fever. Towards the conclusion, the muscles of the face and extremities are often convulsed, while the thumb and fingers are strongly bent inwards, the pulse being imperceptible at the wrist. We need not pursue the details of this last scene. They are too familiar to the practitioner.

The *peculiarities* in this complaint are very deserving of attention, as tending to puzzle the medical attendant. We shall extract a few of these from the text of our author.

"1st. I have seen several instances in which a considerable quantity of water had been collected within the ventricles of the brain, and in which these ventricles had been considerably enlarged, notwithstanding which, there were no peculiar symptoms which indicated its presence."

"2dly. The disease sometimes begins by repeated convulsive fits, or by a spasmodic contraction of the extensor muscles of the head; by which it is forcibly drawn backwards, and kept so until a day or two before death. This symptom was so strongly marked in two cases, that the mothers of the patients recognised the disease in another child, from the peculiar manner in which the head was drawn backwards; so that the disease somewhat resembles tetanus, but the head was not so rigidly drawn back as in that disease, for it might be bended forwards; but as soon as the hand was taken away, the patient drew it back again.

"These convulsive fits were the first symptoms of derangement in the functions of the system which attracted the attention of the parents, and which led them to seek for medical aid.

"Squinting is sometimes observed about the beginning of the disorder; and the pupil is much dilated, and has somewhat of a reddish colour.

"In some cases there is no affection of the pupil during the whole course of the disease.

"The disease sometimes commences by sleepiness.

"Excessive costiveness is on some occasions the primary symptom of this disease."

Sometimes persons affected with hydrocephalus are seized with hemiplegia, and die in the course of a day or two—and, on dissection, three or four ounces of water will be found in the ventricles of the brain. The disease sometimes begins like acute rheumatism, and the symptoms of cerebral affection do not shew themselves till three or four days before death. In some instances, there are no symptoms of irritation, but merely those of compression, as insensibility, coldness of the head and extremities, slowness and irregularity of the pulse and breathing. A day or two before death, Dr. M. has seen little patients recover their senses, take food, and shew symptoms of recovery; but suddenly the phenomena of effusion come on, followed by convulsions and death. Squinting is by no means a constant symptom. There are instances in which the disease has terminated favourably after convulsions, blindness, and delirium, had taken place, and, after the patient had been supposed to be dying. Some patients continue sensible during the whole progress of the disorder, except for a few hours before death.

### Cause of the Effusion.

This, like most other topics of causation, has occasioned great discussion and discrepancy of opinion. According to Quin, there is but one cause—" a morbid accumulation of blood in the vessels of the brain—sometimes proceeding to a degree of inflammation—and generally, but not always, producing an extravasation of a watery fluid." Drs. Rush, Yeats, and Golis, (of Vienna) also consider the disease as the result of inflammation and turgescence. Before subscribing to this hypothesis, which must operate so powerfully on practice, Dr. Monro thinks it necessary to inquire, " whether this disease usually occurs, in persons who are disposed to inflammatory disorders, at or near the meridian of life, when the human body is most liable to suffer from inflammatory diseases." Although several points in the following train of reasoning are defective, if not erroneous, yet Dr. Monro has adduced sufficient evidence to prove the fallacy of that modern doctrine, (to which we lately alluded, when reporting Mr. Lawrence's sentiments in the Medico-Chirurgical Society) which represents all serous effusions into any of the cavities of the body, as invariably the products of inflammation.

" With regard to the first of these points, it may be observed, that hydrocephalus is so rare after puberty, when the constitution is most liable to inflammatory disorders, that Dr. Cullen,* and other writers of eminence, have described it as being peculiar only to infancy. That the disease is rather to be imputed to debility follows from the well-known fact, that hydrocephalus is frequently a disease which may be traced to bad nursing, improper food, dentition, the sequel of the most tedious and debilitating disorders, as hooping-cough and scarlatina. Besides, hydrocephalus is

* " See his Definition of the Disease."

often a disease of the fœtus in utero, which nips the bud before or soon after it is blown; for the child dies soon after birth, after having made a few laborious and convulsive respirations.

"If Dr. Quin's theory had been well founded, hydrocephalus, like an inflammation of the lungs, and other inflammatory complaints, should have been most prevalent amongst robust men during the prime of life, when the human frame is most prone to other inflammations; whereas, it is a disease of infancy, of debility, and very often connected with a scrofulous habit of body. If it be supposed that hydrocephalus is always connected with inflammation of the brain, and that inflammation gives rise to the softening of that organ (which is the favourite opinion of Lallemand, Rostan, and others), in that case, the brain should be found invariably in a softened state, which is not consonant to my observations.*

"Dr. Mills of Dublin has published twelve cases of hydrocephalus, several of which appear to have been connected with inflammation of the brain, but no mention is made of softening of the brain; on the other hand, in one of these, the brain is said to have *been harder* than natural.

"Dr. Hooper, at page 23, in his explanation of his Plate, representing inflammation of the brain, has observed, 'a pulpy state of the part *is now and then met with.*'

"Dr. Watson also has described a case in which the septum lucidum was of an unusual thickness and firmness.

"If hydrocephalus had originated from inflammation, it should have been more frequently the *immediate* consequence of external violence. Whereas Dr. Cheyne observes: 'With extensive opportunities of seeing hydrencephalus, I have *not met one instance* of its having been directly, and I believe only one where *it was indirectly, occasioned by external violence;*' and when (as sometimes happens) it originates from such a cause, the effect does not follow until months or years have elapsed, and when a debilitated action of the blood-vessels has been induced by the violence, just as palsy is sometimes a consequence of external injury, which palsy is followed by dropsy.

"If inflammation of the brain had given rise to this species of hydrocephalus, the attack of the disease should be sudden and well marked, and its course rapid, like to that of phrenitis; whereas the origin of the disease is generally not well marked; indeed, so much so, as often to escape the notice of the parent, and even that of the experienced physician.

"It may here also be observed, that I have made experiments upon several rabbits and pigs, with the view of determining this question, but though I excited inflammation of the brain by trepanning the skull, and cutting off a portion of the dura mater, the effusion of a watery fluid within the head did not follow.

"It is admitted even by those who impute hydrocephalus to an inflammation of the brain, that the symptoms of phrenitis are well marked, whereas those of hydrencephalus are often very obscure; indeed, in some cases, there *is no one symptom indicating the effusion within the head.* I have met with four cases in which a watery fluid was collected within the ventricles of the brain, and in all of these there were none of the symptoms during life which led to the most distant suspicion of water being lodged within the ventricles of the brain.

"If this species of hydrocephalus be owing to an inflammatory state of the brain, there ought to be no distinction as to the symptoms, origin, progress, and consequences of phrenitis and hydrocephalus.

"Inflammation of the brain is thus defined by Dr. Cullen: '*Pyrexia vehemens; dolor capitis; rubor faciei et oculorum; lucis et soni intolerantia; pervigilium; delirium ferox, vel typhomania.*'

"The symptoms of this species of hydrocephalus do not correspond with the above definition.

---

* "Vide Case described at p. 42, in which there were marks of preceding inflammation."

"One of the most striking features of inflammation of the brain, is the state of the pulse; but that character is also awanting, for the state of the pulse is widely different from that of a person afflicted by apoplexy or inflammation of the brain. It is not full, as in the former, or hard, as in the latter. It is no doubt quick, as in other diseases which are the effect of debility; in the same manner as the pulse rises after a great deal of blood has been drawn.

"Beside, no one author who has described the symptoms of phrenitis, has stated *that the pulse becomes slower some time after the commencement of the disorder*; and, on the other hand, bleeding from the eyes, mouth, bladder, and intestines, which are so frequent during phrenitis, have seldom or never been observed to occur during the progress of hydrocephalus. If hydrocephalus depended upon a slight degree of inflammation, the disease would be more frequently cured.

"There is a great difference between the watery fluid which is effused in this species of hydrocephalus, and that in the former most acute species which is connected with inflammation. The latter is turbid, and masses of coagulable lymph shoot through it, and it resembles water accumulated within the abdomen in cases of dropsy of the belly, originating from peritoneal inflammation. Whereas the former is as clear as spring water; and as BELLINI, BOERHAAVE, DU HAEN, MALPIGHI, Drs. WATSON, CARMICHAEL SMYTH, and COINDET remark, the effused fluid does not coagulate by the application of heat or the mineral acids."

The analysis of the hydrocephalic fluid by Dr. Trail, is in support of our author's opinion. At the first tapping, no coagulable lymph was found, as the fluid had not been derived from an inflamed surface; but, after tapping the brain, which operation had probably induced some degree of inflammation, the nature of the effused fluid was materially altered—it contained a certain proportion of coagulable matter.

Dr. M. observes that, an inflammation of the brain is frequently the immediate sequel of insolation, or of external violence. But water in the head is not occasioned by similar causes, nor is the disease more frequent in warm than in cold climates, like phrenitis—nor is it the immediate effect of external violence—nor of trepanning the skull, and afterwards injuring the brain of the animal, a fact which he ascertained by repeated experiments on animals.

"Injuries of the head, no doubt, sometimes give rise to hydrocephalus, but the effect does not immediately follow the cause; *the fluid is not effused until after the lapse of several months or years*, when the vessels have become debilitated, in consequence of the previous over-excitement. (To employ Dr. CHEYNE's own words), 'By calling into play, what, from a good and fortunate management, had hitherto become latent; I mean, *a scrofulous condition of the system, which I have regularly observed to follow a severe accident, and which wonderfully favours the establishment of hydrencephalus*.'

"Some advocates for the opinion that hydrocephalus originates from inflammation of the brain, have imputed the disorder *to a fault in the digestive* organs.

"But a fault of the digestive organs, so far from adding to the vigour of the constitution, produces a very contrary effect, and, by diminishing the vis vitæ, tends to avert or to remove a disposition to inflammation.

"The morbid appearances most frequently discovered on dissection, are generally hostile to the hypothesis, that hydrocephalus acutus is connected with inflammation.

"The pia mater very seldom exhibits, in cases of hydrocephalus, those appearances which, according to the late Mr. J. HUNTER (a very competent judge,) are the genuine marks of inflammation.

"That distinguished surgeon has observed (p. 281), 'When inflammation takes place in parts that have a degree of transparency, that transparency is lessened. This is probably best seen in membranes, such as those membranes that line cavities, or cover bodies in those cavities, such as the pia mater, where, in a natural state, we may observe the bloodvessels to be very distinct. But, when we see the bloodvessels *fuller than common, yet distinct in such membranes, we are not to call that inflammation.*'

"There is no part of the body in which it is so difficult to make the distinction between the presence or absence of inflammation, as in the pia mater.

"The pia mater did not bear, even according to Dr. QUIN, all the characters of genuine inflammation,—it was not thickened,—it did not adhere ultimately to the substance of the brain, as in cases of inflammation of that organ. According to that distinguished pathologist, Dr. BAILLIE, 'when the pia mater is inflamed to a high degree, pus is formed.'

"Dr. QUIN does not state that he discovered pus upon examining the state of the brain of persons cut off by hydrocephalus.

"Besides the thickening of serous membranes, as the dura mater and arachnoid coat, a preternatural adhesion of these generally follows the inflammation of these membranes. The result of my post-mortem examinations is, that, in a very few cases only, there were appearances of preceding active inflammation.

"It may be argued by the advocates for an inflammation of the brain being the invariable cause of hydrocephalus, that the slightest degree of inflammation of that organ, or of its investing membranes, may not be appreciable by our imperfect senses; in the same manner as we cannot suppose (as has lately been stated), that there is no difference in the purity of the air in the centre of London, and that at the top of the Malvern hills of Worcestershire.

"There is no one point more difficult to determine, whether a morbid accumulation of blood really exists; and *granting that it does exist, whether it be connected with inflammation or not?*

"Until the precise caliber of the bloodvessels shall be ascertained, it is quite impossible to say when blood can be said to be accumulated in these vessels. Blood, when accumulated in the bloodvessels, must occasion an enlargement of these, which does not suddenly happen.

"Inflammation is not the necessary concomitant or sequel of an accumulation of blood in the bloodvessels, the eye, for instance, often acquires a red colour, though the pulse be not accelerated, and though there be no degree of pain in the eye or forehead, and no degree of impatience of light, or any other symptom of active inflammation.

"The vessels of that part of the brain which was lowest, were, in *many instances, fuller of blood than those of the uppermost part of that organ, which evidently is connected with the position of the head rather than with inflammation.*

"As the different advocates for the opinion, that hydrocephalus is connected with inflammation, have published very different statements as to the kind and degree of the inflammation, it does not seem to me to be against the rules of evidence, to suppose, as it is so difficult to distinguish the genuine characters of inflammation, that, in some of the instances, *no degree of inflammation had existed.*"

There are other arguments, Dr. M. thinks, which might be urged against the phlogistic doctrine. The same treatment has not been pursued in hydrocephalus as in other inflammations. If it originated in this last, it should be generally removed by the lancet. But local depletion is found more beneficial, and Dr. Quin himself acknowledges "that such patients do not bear large bleedings well." After leeching, Dr. Quin recommended mercury, as an efficacious remedy. Dr. Monro considers mercury as a stimulant and not a sedative—hence it ought to be injurious in purely inflammatory affections. This argument of Dr. Monro is one of the weakest which he has brought forward.

Whether mercury is a stimulant or sedative may be a matter of opinion, or, indeed, of doubt—but that it is injurious in inflammations is negatived by facts and daily observation. The following passage does not quadrate with the modern doctrine which ascribes all dropsical effusions to preceding inflammation.

"Of the identity between hydrocephalus and dropsy there is at least presumptive, if not positive, evidence.

"There is an analogy between the cause of hydrocephalus and dropsical disorders. Hydrocephalus, like dropsy, more frequently originates from debility than from inflammation, and is a frequent disease of infancy,—a period of life when the human frame readily bends under the pressure of every cause, which enfeebles the power of the constitution, as of long continued fever, scarlatina, phthisis pulmonalis, marasmus, measles, whooping cough, and diseases of the liver, spleen, and mesenteric glands.

"Dr. Hamilton senior has justly observed, 'that hydrocephalus often steals slowly on the devoted victim, with symptoms resembling incipient marasmus.'

"Till some better theory is established, it is not unreasonable to suppose, that *the marasmus of which I have treated, may, on some occasions, give rise to hydrocephalus, by impairing the vigour of the constitution, and favouring serous effusion in the ventricles of the brain.*

"It may be added, that Morgagni has mentioned the case of an elderly woman, which strongly illustrates the great influence of debility in producing hydrocephalus; it is said, 'she sank progressively to her grave, as under the pressure of age.'

"The detraction of blood has sometimes occasioned dropsy of the abdomen or chest. The same observation may be extended to hydrocephalus."

The experiments of Dr. Seeds and Dr. Sanders have shewn that the excessive detraction of blood gives rise to effusion of serum within the head.

An impediment to the free return of blood from the venous system is, according to our author, the most frequent cause of hydrocephalus, as, also, of other dropsies. Hence, hydrocephalus is frequently connected with tumours of the brain—with an enlargement of the pituitary gland—tumours in the neck—hypertrophy of the heart—disease of the lungs—"or with any cause which impedes the free return of venous blood, as tumours pressing upon the superior longitudinal sinus, torcular Herophuli, or the jugular veins."

"Hence we meet with the effusion of water within the ventricles of the brain of criminals who, when in perfect health, have been killed by suspension. It is remarkable how soon the effect follows the operation of this cause. I found about half an ounce of water at the basis and within the lateral ventricles of the brain of a criminal, whose body I examined immediately after execution; and in other criminals, after the lapse of a few hours.

"Dr. Kellie of Leith, in his very ingenious paper upon death from cold, has observed, 'The effusion which was discovered within the heads of our subjects, can hardly be regarded as a post-mortem production; nor can it be presumed that it existed previous to their exposure on that night which terminated their existence. The perfect parallelism of the two cases,—their agreement with another case by Quelmalz,—their simultaneous exposure and death on the same night that another individual died under similar circumstances, render such a supposition highly improbable. If this serous effusion were not a post-mortem effect, and if it had no existence previous to the exposure of the individuals, then we must conclude that the whole, or the greater part, was effused in the short interval between their exposure and their death."

A suppression of accustomed evacuations is acknowledged by all writers to be a very frequent cause of dropsy, and it may be extended to hydrocephalus. Dr. Golis, a warm advocate for the inflammatory origin of dropsy, tells us that, "when the urine is unnaturally scanty, the physician ought to be on the watch for an affection of the head." Hydrocephalus is very often connected with scrophula, and is most frequently found in scrophulous families. Dr. Percival, of Manchester, remarked that, out of 22 children who died of hydrocephalus, eleven were manifestly scrophulous.

"The appearances on dissection, in cases of hydrocephalus, scrofula, and dropsy, are similar.

"When water has been accumulated within the ventricles of the brain, scrofulous tumours in the vicinity of the venous sinuses, or in other parts of the brain, are occasionally found, scrofulous tubercles of the lungs or liver, and a scrofulous enlargement of the mesenteric glands. See cases above described, at page 49.

"The analogy between hydrocephalus and dropsy, may be further traced from the effect of the remedies by which both diseases have been occasionally relieved or cured.

"Hydrocephalus is occasionally removed by the same remedies as other kinds of dropsy, though, on account of the peculiar structure of the brain, and the effects of the disease in deranging its structure, the cure is more uncertain.

"From what has been above stated respecting the origin, causes, and appearances on dissection, instead of regarding hydrocephalus as generally connected with inflammation, it is rather to be imputed to scrofula, or to those causes which occasion a derangement in the circulation of blood through the brain, through the bowels of the chest and belly, than to inflammation of the brain, or what has been called Sub-inflammation by authors, and which acts peculiarly on a scrofulous habit."

The prognosis is generally unfavourable, though by no means so invariably so, as some writers would teach. Whytt acknowledged that he never cured a single patient who had those symptoms which certainly denote the disease. Fothergill made a similar confession. The reasons why hydrocephalus is so generally fatal, are ably summed up by Dr. Monro in the following manner.

"1st, Because when water has been accumulated within the brain, the functions of that important organ are, by the pressure of the accumulated fluid, more or less deranged, and the pressure probably occasions a deviation from the healthy structure of those very important parts of the brain which are in the vicinity of the ventricles; to which is to be added the influence of water, when once accumulated, giving occasion to the rapid effusion of more fluid, from its compressure upon the veins proper to the membrane which lines the ventricles, the large vein of GALEN, and the velum interpositum.

"2d, The disease often proves fatal, because it is often connected with an organic disorder of the brain, or of its investing membranes, or with organic disorders in the neck or bowels of the chest or belly, which in many cases cannot be removed.

"3d, Because, if we adopt the opinion of some authors, that it arises from a congestion of blood in the veins, the observations and ingenious experiments of Dr. KELLIE have shewn, that it is very difficult to remove such a congestion. The case detailed in pages 112 and 113 of this Essay, and the experiments of Dr. Seeds, shew that large bleeding is followed by effusion of fluid into the ventricles.

"4th, Whether the softening of the brain be the prelude to the effusion, or the consequence of it, considering the disorganization of the brain thereby produced, it is difficult, probably impossible, to remove it."

Notwithstanding the above propositions, which are but too well supported by the experience of the profession, there are not wanting many instances in which all the more usual symptoms of the disease, already enumerated, have yielded to the efforts of nature, or to the means which the healing art has supplied. Drs. Monro, Percival, Dobson, Rutherford, and many others, concur in this opinion. "There are also a few examples in which the effusion of water has ceased, after the head had attained an extraordinary magnitude."

### TREATMENT.

We have been much more minute in our analysis of the other chapters of the work before us than we shall be on this. The treatment of a disease will vary much according to the pathological notions which the practitioner may have imbibed.

"If the disease be supposed to originate in debility, in laxity of the brain and its vessels, it may possibly be averted,—by avoiding cold,—all vicissitudes of the weather,—and every means by which the bodily strength may be impaired,—and by endeavouring to improve and invigorate the constitution, by generous diet, wine, and the keeping the bowels regular,—and by removing irritation, and the irregular action of the chylo-poietic viscera,—by warm clothing, and moderate and daily exercise in a pure atmosphere.

"By adopting such a mode of treatment, I think I have had the satisfaction of averting the disease.

"But, on the other hand, should the disease be supposed to proceed from the over-excitement of the vessels of the brain, and if inflammation be the primary cause, and the effusion merely the effect, an attempt should be made to remove that state by the general and topical detraction of blood, low diet, by refrigerant applications to the head, by purgatives, and by setons applied to the neck, by spices and blisters, and by avoiding all such causes as induce plethora.

"In my description of the symptoms of the subacute hydrocephalus, I have endeavoured to point out to my reader, that a derangement of the functions of the alimentary canal gives rise to the earlier symptoms of hydrocephalus. The patient's stomach is disordered; the abdomen is often tense, and painful; the stools are like clay, of a variegated colour, and of a very offensive smell; hence the necessity of evacuating the contents of the intestines by calomel and other purgatives.

"I have stated torpor of the intestines to be a consecutive symptom; and this merits peculiar notice, as indicating a derangement in the functions of the brain, in which case, a large blister should be applied over the head.

"Calomel, combined with James's powder, is of great use in restoring the healthy functions of the bowels."

Fothergill, Rush, Cheyne, and Carmichael Smyth, have highly recommended the daily use of the more powerful cathartics, as gamboge, colocynth, scammony, combined with calomel, and these in large doses, on account of the torpor of the bowels.

"In general, children bear calomel better than adults. If calomel be given in an over dose, it produces colic and severe diarrhœa, and sometimes inflammation of the intestines. A grain may be given every third hour to a child of a year old, and the dose is to be repeated until a free evacuation has been produced. But should the child have acute pain in the belly, the medicine must be given up, for if more be given, an inflammation of the intestines will probably follow. I have given three grains of calomel, three or four times a-day, to children of ten or twelve years of age, afflicted by this disorder; and it did no more than keep the bowels open, or, at most, produced only three or four stools."

Dr. M. informs us that, by the application of a large *blister*, composed of tartar emetic and wax ointment, to the head, and the use of calomel, combined with James's powders, he has cured the disease, not in one, but in several instances. Dr. Rutherford, his colleague at the Dispensary, adopted the same plan, and has stated to Dr. Monro that he was equally successful. Dr. M. was first led to the employment of James's powder, from the recommendation of Dr. Cheyne, who speaks highly of its efficacy in this dangerous disease. "If there be pain and tenderness in the region of the liver, the patient will derive much benefit from the application of leeches to the abdomen." Dr. M. prefers one large *blister* of tartar emetic to a succession of small ones composed of cantharides. The latter occasion strangury and much irritation. But the fact is, that the operation of the one is very different from that of the other. The eruption of pustules, and the production of vesication, are very dissimilar processes, and effect very different actions in the animal economy. We will not renew the discussions respecting the efficacy of mercury in this disease. If we are acquainted with the sentiments of writers and practitioners generally, on this point, we will say that the utility of the medicine under consideration, is put beyond the possibility of cavil. It is true that some have recommended calomel in large doses—some in small. Perhaps there is little difference in the ultimate effects of both plans. When the head is affected, there is usually such torpor of the bowels, that large doses of medicine produce no more action in the intestinal canal than much more diminutive quantities. Hence the great deception into which practitioners may be led. Dr. Monro has given us the following document from his father, with which we will conclude this analysis.

"After mentioning twenty-two cases of this disorder, in which he had unsuccessfully employed mercury, he states:

"'As in the greater number of the above cases, the disease had made considerable progress before I was called; and, as most of the patients survived but for a short time thereafter, the effects which the mercury may have, if given on the first appearance of the symptoms, are by no means fully determined. And, as I have repeatedly found, in other dangerous species of the natural encysted dropsy, particularly in hydrothorax and ascites, that mercury combined with squills or other diuretic medicines, in such quantity as to salivate in a slight degree, contributed much to the relief or cure of the patient, I would recommend the further trial of it in hydrocephalus. At the same time, considering the importance, sensibility, and delicate texture of the parts which are affected, and total failure in the cases I have described, I cannot help suspecting that several late writers are much too sanguine in their expectation of removing hydrocephalus by the use of mercury.'"

We must now close this article, promising to furnish our readers with some important information from the same work, respecting certain pathological conditions of the brain, in one of our succeeding numbers.

We have restricted ourselves, in this paper, almost entirely to the humble, though not the less useful, labour of analysis. But the talented author will excuse us, if we venture to dissent a little from him, as to the part which inflammation takes in the serous effusion which is usually found in the disease under consideration. We have often expressed our opinion, that it was too much the fashion to consider phlogosis as the root and branch of the effusion. But we cannot, consistently with our own observations, go the length which Dr. Monro has gone, in the opposite view of the pathology of hydrocephalus. We are convinced that, in a great majority of cases, the effusion which we find in the heads of infants who die with the common symptoms of hydrocephalus, is the result of an inflammatory process more or less acute—and, consequently, that our remedial measures should generally embrace local depletion from the head—especially in the early stage of the disease. At the same time, we are glad to have the authority of Dr. Monro, in support of a doctrine which we have long advocated—namely, the possibility of watery effusion from other causes than inflammation.

Although we mean to return to Dr. Monro's work again, we cannot take leave of it, without expressing our sincere esteem for the amiable and talented author. The small volume which he has now published, abounds in rich materials, without the slightest tincture of wild hypothesis—arrogance—presumption—or the prevailing sin of the day—censure of the opinions or practice of his brethren. Dr. Monro is a worthy representative of the old school of amenity, liberality, and true love of science! If primus et secundus Monro could start from their marble cerements, and view the distractions of medical society at the present moment—if they could read the malevolent and ignorant effusions of a SCOTUS, indited in the Intellectual City—they would, doubtless, join in the doleful verse of their countryman, Smollet—

"Mourn, hapless Caledonia, mourn—
Thy banished peace—thy laurels torn!"

